# Application of a multivariate approach to the study of chemometric and sensory profiles of cookies fortified with brewers’ spent grain

**DOI:** 10.1007/s13197-024-06064-3

**Published:** 2024-08-23

**Authors:** Antonietta Baiano, Anna Fiore, Mariacinzia Rutigliano, Barbara la Gatta

**Affiliations:** Dipartimento di Scienze Agrarie, Risorse Naturali e Ingegneria (DAFNE), University of Foggia, Via Napoli, 25, Alimenti, Foggia, 71122 Italy

**Keywords:** Antioxidants, Brewers’ spent grain, Dietary fibre, Functional cookies, Sensory quality, Sustainable Food production

## Abstract

This work was aimed to investigate the effects of three factors on cookie quality: brewers’ spent grain (BSG) composition [65% malted barley and 35% of unmalted durum (DA) or soft (RI), or emmer (EM) wheats]; geographical origin of the cereals used in brewing (Daunia or Salento); and percentages of BSG in cookie formulation (30 or 40%). A control made of 100% Manitoba flour was produced. Statistical analyses were performed to evaluate the effects of those factors (Analysis of Variance), the possibility to distinguish the various types of cookies (Principal Component Analysis), and the relationships among variables (Pearson Correlation Analysis).The single and interactive effects of the three factors were significant for almost all variables. Cookies with 40% EM spent grains showed the highest ash, dietary fibre, and total phenolic contents but cookies with 30% DA or RI spent grains received the highest overall quality scores due to the higher intensity of their fresh baked flavour and their lower hardness and fibrousness. Based on the nutritional and sensory characteristics, cookies fortified with RI and DA were the best to consume. Although few physicochemical differences can be attributed to geographical origin, a slightly higher overall sensory score was assigned to those produced with Salento cereals. Principal Component Analysis showed a clear separation between the control made of 100% Manitoba flour and the group of fortified cookies. Among the latter, the cookies produced with RI and DA spent grains were indistinguishable from each other due to their similar quality characteristics.

**Supplementary Information:**

The online version contains supplementary material available at 10.1007/s13197-024-06064-3.

## Introduction

Bakery products are one of the potential choices for the addition of functional ingredients since they are widely consumed, are available at generally accessible cost, can be stored for long time at room temperature, and contribute significantly to the daily intake of energy, carbohydrate, protein, B group vitamins, and minerals. However, they are not carriers of phenolic compounds when produced with refined flours (Papageorgiou et al. 2018). Among bakery products, cookies are an ideal vehicle for the delivery of micronutrients and other bioactive compounds for additional reasons such as consumer appreciation and elevated practicability for daily diets growing consumption (Pinto et al. 2023).

A joined definition of functional food is that it is a healthy food similar to the conventional food counterpart, consumed within a usual diet, and claimed to exert physiological health benefits beyond the nutritional function. Among the functional compounds, polyphenols and soluble and insoluble fibres deserve to be mentioned. Sources of functional ingredients are represented by herbal plants, spices, animal, seafoods, and microorganisms. The exploration of further sources of bioactive compounds is an advantageous research field, due to the increasing importance of functional foods for the world’s population. A step forward has been made by investigating the possibility of producing healthy foods from sustainable food systems, with the dual purpose of meeting the demand of functional foods and encouraging environmental sustainability (FAO & WHO, 2019). One step in this direction is the enrichment of foods with functional ingredients from agri-food waste and agro-industrial by-products (Melini et al. 2020). The enrichment of cereal-based products by agri-food waste increases their content in phenolic compounds, vitamins, fibres but can have a negative impact on technological and sensory quality (Gómez and Martinez 2018). Valorisation of agro-industrial by-products may have substantial effects on environmental harm reduction, economic growth, and improvement of human health, particularly used as global functional ingredients.

Brewers spent grains is quantitatively the main by-product of breweries, since it accounts for 40% of beer manufacturing waste equivalent to an annual production of 39 million tons (Garcia-Garcia et al. 2019; Lukinac and Jukic 2022).

The BSG composition has been deeply studied by several researchers (Cooray and Chen 2018; Nocente et al. 2019) and can differ due to the type of beer ingredients, brewing process, and spent grain drying methods. Dried BSG has the following mean composition: 5–8% moisture; 2.7-5% ash; 14.5–30% protein nitrogen (from hordein, glutelin, globulin, and albumin); 0.43–2.17% starch; 8-34.82% fat (mainly triglycerides); minerals such as phosphorus, calcium, sulphur, magnesium, and potassium; dietary fibre, β-glucans; phenolics (mainly protocatechuic, caffeic, *p*-coumaric and ferulic acids, catechin, and derivatives).

Due to their composition, BSGs can be conveniently included in cereal-based products such as bread, extrudates, cookies, snacks, crackers, and pasta. Although BSG improves the nutritional value of the end products, it also affects processing and the physicochemical, mechanical, and sensory quality of the final products, especially in the case of leavened products and pasta in which the formation of a regular gluten network is hindered by the interference of the BSG fibres. Regarding the incorporation of BSG in biscuits or cookies, it has been stated the occurrence of a spread ratio reduction as negative effect that, however, can be counterbalanced by the mechanical action exerted during the mixing process, thus allowing the formation of a better protein network (Odeseye et al. 2020). More in depth, the effect on the spread ratio depends on the amount of BSG added, because a low quantity increases this parameter while a greater addition reduces it (Ajanaku et al. 2011; Heredia-Sandoval et al. 2020). Regarding the nutritional and health impacts of BSG-enriched cereal products, supplementation increases fat, mineral, protein, antioxidant, and fibre content and exerts antihyperglycemic effects (Heredia-Sandoval et al. 2020; Uchegbu 2016). Concerning the influence of BSG addition on cookie sensory quality, the maximum substitution level tolerated without detrimental effects on taste and texture varies from a research to another, ranging from 6% (Ajanaku et al. 2011) to 20% (Uchegbu 2016), probably as a result of the addition of other ingredients or the specific characteristics (composition, origin, any pre-treatments undergone) of BSG itself that in turn depend on the history of spent grains.

As seen, the effect of the amount of BSG in food formulation is the most investigated variable, but our aim was to analyse the effects of other parameters that have been treated only in few studies concerning BSG enriched bread (Baiano et al. 2023). For this reason, our study was intended to increase the knowledge of the single and interactive effects of BSG composition, geographical origin of the starting cereals, and percentages of BSG in formulation on cookie quality attributes such as content of nutraceutical compounds and sensory characteristics.

## Materials and methods

### Production of BSG flour

Barley malt cv. Fortuna was supplied by Agroalimentare Sud (Melfi, Potenza, Italy). The unmalted common wheat (*Triticum aestivum*) cv. Risciola, durum wheat (*Triticum durum*) cv. Dauno III, and emmer (*Triticum dicoccum*) were obtained from plants born from seeds selected by CREA-CI Research Centre for Cereal and Industrial Crops (Foggia, Italy) and cultivated under the same conditions in two areas of Puglia region, namely Daunia and Salento as described in Baiano et al. (2023). These two locations were chosen for various reasons: they are both suited to the production of cereals for the malting and brewing industries; the same barley and wheat varieties are cultivated in both areas; both produce the raw material for the malthouse which supplied the malt used in the research. BSGs were recovered as by-products of the mashing step, during the brewing operations concerning the production of six types of craft Belgian white beers starting from cereal mixtures made of 65% barley malt and 35% of one of the unmalted cereals (common wheat, durum wheat, or emmer) cultivated in Daunia or in Salento, two areas located in the northern and southern parts of Puglia, respectively. Six types of BSG - namely RID, RIS, DAD, DAS, EMD, EMS – were dried, ground, and stored as described by Baiano et al. (2023) that, in the same paper, highlighted their physical and chemical characteristics.

### Formulations and production of the functional cookies

The following ingredients were used in cookie formulations: the six BSG flours; Manitoba common wheat flour type 0 (COOP, Casalecchio di Reno, Italy); brown sugar (Friessinger Mühle, Bad Wimpfen, Germany); extra-virgin olive oil (Pazienza, Foggia, Italy), fresh whole eggs (Le Naturelle, Santa Maria In Fabriago, Italy); baking powder and vanillin (Pane degli Angeli-Cameo, Desenzano del Garda, Italy); organic lemon zest, recovered on a local fruit and vegetable market; cinnamom (Drogheria and Alimentari S.p.A., Scarperia e San Piero, Italy).

**Fig. 1:**
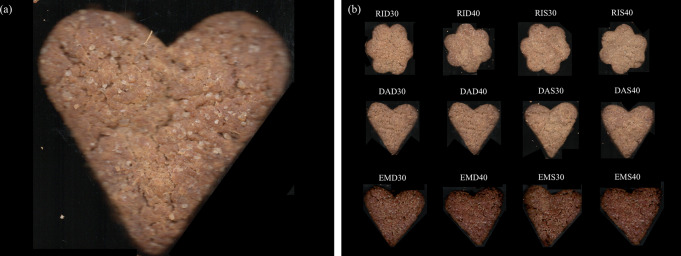
Control cookies (**a**) and cookies (**b**) enriched with brewers’ spent grain

Thirteen types of cookies were produced according to the formulations reported in Table 1: a control made of 100% Manitoba common wheat flour type 0 (CS); and twelve functional cookies, obtained by replacing the Manitoba flour with two different amounts (30 or 40%) of each of the BSG flours (Fig. 1). Cookies were produced by introducing all the ingredients in the bowl of a Thermomix TM6 (Vorwerk, Wuppertal, Germany) and kneading for 20 s at 4.5 speed. The obtained doughs were wrapped in transparent film, placed in the fridge to harden for 30 min, and then rolled out into a sheet of about 6 ± 1 mm thick. The cookies were cut out with regular shape cutters and baked at 180 °C for 20 min.

**Table 1:** Ingredients used in cookie production (g/100 g formulation)

Cookies	BSG flour^1^	Manitoba flour	Brown sugar	EVOO	Whole eggs	Baking powder	Lemon zest	Vanillin	Cinnamom
CS	0 (0%)	58	13.9	10.6	15.0	1.8	0.5	0.1	0.1
RID30	17.4 (30%)	40.6	13.9	10.6	15.0	1.8	0.5	0.1	0.1
RID40	23.2 (40%)	34.8	13.9	10.6	15.0	1.8	0.5	0.1	0.1
RIS30	17.4 (30%)	40.6	13.9	10.6	15.0	1.8	0.5	0.1	0.1
RIS40	23.2 (40%)	34.8	13.9	10.6	15.0	1.8	0.5	0.1	0.1
DAD30	17.4 (30%)	40.6	13.9	10.6	15.0	1.8	0.5	0.1	0.1
DAD40	23.2 (40%)	34.8	13.9	10.6	15.0	1.8	0.5	0.1	0.1
DAS30	17.4 (30%)	40.6	13.9	10.6	15.0	1.8	0.5	0.1	0.1
DAS40	23.2 (40%)	34.8	13.9	10.6	15.0	1.8	0.5	0.1	0.1
EMD30	17.4 (30%)	40.6	13.9	10.6	15.0	1.8	0.5	0.1	0.1
EMD40	23.2 (40%)	34.8	13.9	10.6	15.0	1.8	0.5	0.1	0.1
EMS30	17.4 (30%)	40.6	13.9	10.6	15.0	1.8	0.5	0.1	0.1
EMS40	23.2 (40%)	34.8	13.9	10.6	15.0	1.8	0.5	0.1	0.1

^1^ the percentage of replacement of Manitoba flour with BSG is reported within brackets

### Physical and chemical analyses

>The cookie chromatic characteristics, determined as described by Baiano et al. (2023), were expressed according to the coordinates of the colour space defined by the International Commission on Illumination: L*, lightness/brightness from black to white on a scale of zero to 100; a*, from negative (green) to positive values (red) and b*, from negative (blue) to positive values (yellow), both without specific numeric scale limits.

Moisture and ash contents of ground cookies were quantified according to the AACC Methods 44-15.02 and 08-01.01 (2012), respectively, and expressed as %.

Soluble and insoluble dietary fibres were determined according to AACC Methods 32-05.01 and 32-21.01 (2012) using the K-TDFR-200 A Megazyme kits (Neogen Europe Ltd., Ayr, Scotland) and expressed as %.

The cookie proteins were extracted as described by Baiano et al. (2023) and submitted to electrophoretic and chromatographic analyses according to la Gatta et al. (2017). Electrophoretic separation (SDS-PAGE) was performed through a Mini-PROTEAN Tetra system electrophoresis cell (Bio-Rad, Hercules, CA, USA). The polymeric components of cookie proteins were analysed using a two-step extraction procedure using a Biosep SEC-S4000 column (300 × 7.8 mm, Phenomenex, Torrence, CA, USA). The results were expressed as SDS-Extractable and SDS-Unextractable total peaks area and as unextractable polymeric proteins (UPP%) according to Kuktaite et al. (2004).

The phenolic fraction was analysed to evaluate the functional potential of the enriched cookies. The phenolic component was extracted from previously ground cookies according to Gandolpho et al. (2021), analysed using the Folin–Ciocalteu reagent, and expressed as mg of gallic acid/g of dry weight. The concentrations of each compound were determined by a HPLC-DAD system (Agilent 1100 Liquid Chromatograph, Santa Clara, CA, USA) equipped with a 100 × 4.6 mm × 3 μm RP-C18 Gemini column (Phenomenex, Aschaffenburg, Germany), according to Aliakbarian et al. (2011). The identification of phenolics was made by comparing their retention times and spectra with those of 18 standard compounds. Their quantification (µg/g dw) was obtained by comparing their peak areas with those of standard curves at two wavelengths: 280 nm (gallic acid, 4-hydroxybenzoic acid, vanillic acid, caffeic acid, syringic acid, chlorogenic acid, ferulic acid, rosmarinic acid, epicatechingallate, rutin, resveratrol, quercetin and kaempferol) and 320 nm (catechin, epicatechin, epigallocatechin, *p*-coumaric acid, and sinapic acid).

The antioxidant activity values of the extracts were measured using 2,2-diphenyl-1-picrylhydrazyl (DPPH) free radical scavenging assay (Brand-Williams et al. 1995) and the results were expressed as both mg of Trolox (6-hydroxy-2,5,7,8-tetramethylchroman2-carboxylic acid)/g of dry weight and % remaining DPPH.

### Sensory descriptive analysis

A panel of 12 trained judges (6 females and 6 males) between 25 and 65 years of age, experienced in the sensory evaluation of baked foods, carried out a Quantitative Descriptive Analysis (QDA) as described by Baiano et al. (2023). The profile sheet included 1 visual (colour intensity), 6 olfactory (overall flavour intensity, freshly baked cookies, wheat, malty, toasty, and hazelnut/dried fruits), 2 gustatory (sweetness, bitterness), and 5 tactile (astringency, hardness, crunchness, fibrousness/graininess, and friability) attributes to evaluate on numeric category scales. The attribute definitions were supplied to the panellists that were also asked to evaluate the overall quality of each sample, taking into account all the sensory attributes valuated. The panellists rated the intensity of each parameter on a 0–9 scale. Judges rinsed their mouths between samples with natural water. Samples were completely evaluated in a tasting session.

### Statistical analysis

Each analysis was replicated at least three times and then the averages and the standard deviations were calculated, using Excel software V. 14.0.0 for Mac. A three-way ANOVA followed by LSD test (*p* < 0.05) was applied to highlight the single and interactive effects of geographical origin, BSG composition, and percentages of BSG in formulation on physical, chemical, and sensory characteristics of cookies. The Principal Component Analysis (PCA) was applied to verify the possibility of distinguishing the various types of cookies from each other and with respect to the control samples according to physical, chemical, and sensory data. Pearson correlation analysis (*p* < 0.05) was performed to examine the relationships between cookies variables. The results are included in the supplementary material namely Table S1, where the highly correlated variables (R > |0.70|) are reported in red character. ANOVA, PCA and Pearson correlation analysis were carried out using the statistical package Statistica for Windows V. 7.0. (Statsoft, Tulsa, OK, USA).

## Results and discussion

### Physical and chemical analysis of the BSG-enriched cookies

The physical characteristics and chemical composition of the experimental products are shown in Table 2. Concerning colour, our results confirmed the findings of previous researches (Odeseye et al. 2020) i.e. that the substitution of Manitoba flour with BSGs made more intense the cookie colour, with statistically significant decreases of lightness, a*, and b*, as a consequence of the additive effects of the BSG dark colour to the typical baking reactions (Maillard reaction, sugar caramelization, starch dextrination). No significant differences among the values of colorimetric indices could be attributed to the geographical origin of the starting grains while, as aspected, colour decreased as the BSG substitution increased. Concerning the effects of BSG composition, cookies made with EM flours showed a* values comparable to that of DA flour – probably due to their high carotenoid content (Shewry and Hey 2015) - together with a more intense brown colour and a lower luminosity than cookies enriched with RI and DA flours that we supposed dependent on an extensive enzymatic browing related to the highest total phenolic content of EM cookies (Ou et al. 2019).

**Table 2:** Single and interactive effects of geographical origin, BSG composition, and amount of BSG in formulation on physical characteristics, total phenolic content, and antioxidant activity of cookies

Cookies	Colorimetric Indices			Moisture %	Ash %	Dietary Fibre			Extractable	Unextractable	sUPP%		TPC (mg gallic acid/g dw)	Antioxidant Activity			
	L*	a*	b*			TDF	SDF	IDF						% remaining DPPH	Trolox (mg/g dw)		
Interactive effects of geographical origin, BSG composition, BSG % in formulation																	
CS	58.3 ± 6.5^d^	21.7 ± 1.5^g^	49.7 ± 3.5^d^	3.44 ± 0.64ef	1.08 ± 0.01^a^	2.89 ± 0.03^a^	1.81 ± 0.01^h^	1.08 ± 0.02^a^	35757.3 ± 151.8^m^	17429.5 ± 28.2^k^	38.0 ± 0.6^f^		1.98 ± 0.05^a^	81.2 ± 2.2^i^	3.0 ± 0.4^a^		
RID30	55.0 ± 3.0^cd^	15.7 ± 1.2^ab^	43.7 ± 1.5^bc^	2.76 ± 0.65^be^	1.32 ± 0.02^c^	11.50 ± 0.03^e^	1.58 ± 0.02^e^	9.92 ± 0.02^d^	20503.0 ± 466.3^b^	10613.4 ± 45.4^a^	35.1 ± 1.0^de^		4.38 ± 0.69^b^	77.1 ± 0.1^hi^	3.6 ± 0.0^ab^		
RID40	53.3 ± 3.1^ac^	17.7 ± 0.6^cd^	40.7 ± 1.5^ab^	1.29 ± 0.11^a^	1.34 ± 0.01^d^	13.40 ± 0.07^g^	1.69 ± 0.02^g^	11.71 ± 0.07^h^	29925.0 ± 227.5^k^	11851.5 ± 67.5d	32.4 ± 0.2^c^		4.59 ± 0.69^b^	68.6 ± 1.2^ce^	4.8 ± 0.2^d^		
RIS30	56.0 ± 2.0^cd^	15.0 ± 0.0^a^	42.0 ± 0.0^ac^	2.41 ± 0.35^bc^	1.25 ± 0.01^b^	11.32 ± 0.02^d^	1.27 ± 0.02^c^	10.05 ± 0.02^e^	26949.6 ± 59.8^i^	10981.4 ± 110.0^b^	27.1 ± 1.2^a^		5.31 ± 0.21^b^	66.1 ± 9.0^bc^	5.0 ± 1.3^de^		
RIS40	52.3 ± 1.5^ac^	16.0 ± 1.0^ac^	39.7 ± 0.6^a^	2.19 ± 0.1^5b^	1.36 ± 0.02^d^	11.77 ± 0.03^f^	0.56 ± 0.01^a^	11.21 ± 0.02^f^	27178.6 ± 151.8^i^	13106.9 ± 31.6^g^	32.2 ± 0.8^bc^		4.36 ± 0.43^b^	73.9 ± 0.5^fh^	3.9 ± 0.1^bc^		
DAD30	52.3 ± 2.5^ac^	18.7 ± 0.6de	41.7 ± 0.6^ac^	1.23 ± 0.23^a^	1.38 ± 0.00^e^	10.93 ± 0.02^c^	1.71 ± 0.01^g^	9.23 ± 0.01^c^	22280.9 ± 274.3^c^	12284.5 ± 132.3^e^	35.6 ± 0.5^de^		4.83 ± 0.85^bc^	71.9 ± 0.5^e.g.^	4.3 ± 0.1^bd^		
DAD40	54.7 ± 0.6^bd^	19.7 ± 0.6e^f^	44.0 ± 0.0^c^	3.23 ± 0.56^df^	1.50 ± 0.01^f^	14.04 ± 0.02^k^	1.91 ± 0.01^i^	12.13 ± 0.01^i^	23693.1 ± 133.7^e^	12238.9 ± 53.8^e^	27.6 ± 1.6^a^		5.39 ± 1.04^bc^	70.1 ± 0.8^cf.^	4.7 ± 0.1^de^		
DAS30	54.3 ± 1.5^bd^	18.0 ± 1.0^de^	42.0 ± 1.0^ac^	2.93 ± 0.88^cf.^	1.26 ± 0.02^a^	10.21 ± 0.02^b^	1.57 ± 0.01^e^	8.64 ± 0.01^b^	24798.6 ± 239.3^g^	11983.0 ± 74.9^d^	32.5 ± 2.1^c^		5.00 ± 0.63^bc^	75.5 ± 1.2^gh^	3.9 ± 0.2^bc^		
DAS40	52.0 ± 1.7^ac^	18.3 ± 0.6^de^	41.7 ± 1.2^ac^	3.63 ± 0.41^f^	1.39 ± 0.00^d^	13.54 ± 0.02^h^	1.96 ± 001^k^	11.58 ± 0.01^g^	24396.7 ± 132.4^f^	12849.7 ± 115.0^f^	35.9 ± 1.0^e^		5.17 ± 0.79^bc^	67.0 ± 3.4^cd^	5.3 ± 0.6^ef^		
EMD30	50.0 ± 2.0^ab^	17.3 ± 0.6^bd^	39.0 ± 1.7^a^	2.75 ± 0.01^be^	1.35 ± 0.02^d^	13.85 ± 0.02^i^	1.40 ± 0.01^d^	12.45 ± 0.01^k^	31417.8 ± 111.2^l^	14756.8 ± 192.00^i^	33.0 ± 0.1^c^		5.13 ± 0.84^bc^	71.4 ± 0.5^dg^	4.6 ± 0.1^ce^		
EMD40	49.3 ± 2.1^a^	19.0 ± 1.0^df^	39.0 ± 3.0^a^	1.36 ± 0.18^a^	1.51 ± 0.01^f^	15.32 ± 0.02^l^	1.23 ± 0.02^b^	14.09 ± 0.01^l^	22964.0 ± 195.2^d^	11654.4 ± 140.5^c^	33.9 ± 1.1^cd^		5.43 ± 1.07^bc^	59.6 ± 0.6^a^	6.4 ± 0.1^g^		
EMS30	52.7 ± 2.1^ac^	20.7 ± 0.6^fg^	41.7 ± 1.2^ac^	2.56 ± 0.30^bd^	1.36 ± 0.01^d^	16.75 ± 0.02^n^	1.39 ± 0.01^d^	15.36 ± 0.01^n^	26298.1 ± 164.0^h^	14380.4 ± 91.9^h^	30.4 ± 1.5^b^		5.92 ± 0.03^c^	70.7 ± 1.4^cf.^	4.7 ± 0.2^de^		
EMS40	49.0 ± 3.6^a^	18.0 ± 2.6^de^	39.0 ± 4.0^a^	3.43 ± 0.13^ef^	1.51 ± 0.01^f^	16.68 ± 0.02^m^	1.60 ± 0.01^f^	15.08 ± 0.01^m^	18521.9 ± 70.6^a^	10703.1 ± 24.1^a^	38.6 ± 1.5^f^		5.38 ± 0.30^bc^	61.9 ± 1.3^ab^	5.8 ± 0.30^fg^		
*Significance*	*	*	*	*	*	*	*	*	*	*	*		*	*	*		
Single effect of geographical origin																	
D(aunia)	52.4^a^	18.0^a^	41.3^a^	2.10^a^	1.40^b^	13.17^a^	1.59^b^	11.59^a^	25130.5^b^	12233.2^a^	32.93^a^		4.96^a^	69.8^a^	4.74^a^		
S(alento)	52.7^a^	17.7^a^	41.0^a^	2.86^b^	1.35^a^	13.38^b^	1.39^a^	11.99^b^	24690.6^a^	12334.1^b^	32.77^a^		5.19^a^	69.2^a^	4.79^a^		
*Significance*	ns	ns	ns	*	*	*	*	*	*	*	ns		ns	ns	ns		
Single effect of BSG composition																	
RI	54.2^b^	16.1^a^	41.5^b^	2.16^a^	1.32^a^	12.00^a^	1.27^a^	10.72^b^	26139.0^c^	11638.3^a^		31.70a	4.66^a^	71.42^b^	4.34^a^		
DA	53.3^b^	18.7^b^	42.3^b^	2.76^b^	1.38^b^	12.18^b^	1.79^c^	10.39^a^	23792.3^a^	12339.0^b^		32.89^b^	5.10^ab^	71.13^b^	4.56^a^		
EM	50.2^a^	18.7^b^	39.7^a^	2.53^b^	1.43^c^	15.65^c^	1.40^b^	14.25^c^	24800.4^b^	12873.7^c^		33.96^c^	5.47^b^	65.9^a^	5.38^b^		
*Significance*	*	*	*	*	*	*	*	*	*	*		*	*	*	*		
Single effect of BSG % in formulation																	
0	58.3^b^	21.7^b^	^49.7b^	3.44^b^	1.08^a^	2.89^a^	1.81^b^	1.08^a^	35757.25^c^	17429.50^c^		37.96^c^	1.98^a^	81.2^c^		3.03^a^	
30	53.4^a^	17.6^a^	41.7^a^	2.44^a^	1.32^b^	12.43^b^	1.49^a^	10.94^b^	25374.63^b^	12499.91^b^		32.28^a^	5.09^b^	72.1^b^		4.36^b^	
40	51.8^a^	18.1^a^	40.7^a^	2.52^a^	1.43^c^	14.13^c^	1.49^a^	12.63^c^	24446.51^a^	12067.38^a^		30.42^b^	5.06^b^	66.8^a^		5.16^c^	
*Significance*	*	*	*	*	*	*	*	*	*	*		*	*	*		*	

In column, different letters indicate significant differences at *p* < 0.05 by LSD multiple range test the asterisks indicate significant differences at *p* < 0.05 by LSD multiple range test ns: not significant

The mean moisture content, ranging from 1.36% (EMD40) to 3.63% (DAS40), was significantly influenced by all investigated parameters. The geographical origin of grains affected this parameter, with significantly higher values in S cookies. The higher moisture of S cookies referred to the higher moisture of the corresponding spent grains and cereals. Although the cereals produced in Salento were obtained under the same agronomic practices of those produced in Daunia, the two places undergo different weather conditions with greater rainfall in Salento than in the Dauna plain (Cotecchia et al. 2014). Moisture decreased with addition of BSG flour (already at 30% substitution level), as a result of the lower water contents of BSG flours (2.9–3.4%) respect to Manitoba flour (~ 15.0%) but also depended on BSG composition, with RI cookies showing the lowest water content.

The ash content was in range 1.08% (CS)-1.51% (EMD40 and EMS40) and showed higher values in samples produced with grains coming from Daunia. As for BSG-enriched bread (Baiano et al. 2023), BSG fortification significantly increased the ash content of cookies and the greatest ash amount was contributed by the EM type, followed by DA, and RI. Ash content also increased with the percentage of substitution.

BSG are considered as a source of dietary fibre for humans and their use has been suggested as a way to prevent undesirable increase of plasma lipids. According to Gidley and Yakubov (2019), soluble fibre increases the ‘viscosity’ of digesta thus reducing gastric emptying and slowing nutrient absorption while the insoluble fraction promotes the softening of digesta and support regular bowel movements. For this reason, we decided to quantify both the total fibres and the extent of each of the two fractions. The amounts of each of the two fractions in the cookies were significantly influenced by the three factors studied. First of all, the only samples in which SDF prevailed were the control cookies (62.5%) while in the BSG-added cookies that fraction ranged from 4.7% (RIS40) to 15.6% (DAD30) of the total dietary fibre. This means that BSG provided essentially insoluble fibre (EM 31.4%; RI 27.2%; DA 22.8%) since most of the water-extractable and soluble compounds were released during the mashing stage of the brewing process (Baiano et al. 2023). However, if the objective was to provide a greater quantity of soluble fibre through the BSG, they should be previously treated with cellulolytic enzymes. As an example, Wang et al. (2023) highlighted the possibility to submit BSG to a solid state fermentation with *Rhizopus oligosporus* (a filamentous fungus able to release enzymes such as cellulase, endocellulase, hemicellulase, xylanase and polygalacturonase that break down the fibres) to increase the soluble fraction. TDF and IDF showed the same trend, with the lowest and the highest amounts detected in control and EMS30 cookies, respectively. The geographical origin exerted significant effects on TDF/IDF, higher in products made with BSG from Salento, and SDF, higher in cookies made with BSG from Daunia, as already highighted by Baiano et al. (2023). TDF and IDF were higher in cookies enriched with EM spent grains while SDF was higher in DA cookies. Finally, TDF and IDF increased with the amount of brewers’ spent grains in cookies formulation while SDF showed an opposite behaviour.

Regarding protein size distribution, BSG-enriched cookies showed an unusual chromatographic profile (Figure S1), since they did not contain evident peaks in the range of high molecular weight proteins but only a slight raising of the profile (including peak 1 and 2) - for both the control and the BSG-enriched cookies - and two peaks in the range of monomeric proteins (peaks 3 and 4). As expected, the cookie protein extractability (mean peak area of extractable fraction < 35,757) was lower than that of Manitoba flour (mean peak area 246,702) because the baking process promoted a protein aggregation. The significantly highest total peak area for both the fractions (extractable and unextractable) were quantified in control cookies, while EMS40 cookies showed the lowest values both for the extractable fraction and the unextractable one (togther with RID30). The different extractability rate was due to the different characteristics of the grains (single effect of BSG composition), topic widely discussed by Baiano et al. (2023), and to the different and unpredictable arrangement of the protein network after processing. Environment also contributed to the differences observed, with the highest extractable fraction in samples coming from Daunia and the highest unextractable fraction observed in samples from Salento. Since the same agronomic practices have been applied in the two areas, these differences could be due to weather conditions (Noor Hasniza et al. 2014). Both fractions decreased as the BSG substitution level increased. Since the polymeric fraction elution gave rise to a slight raising of the chromatographic profile in the elution range of small polymeric proteins, we decided to rename the %UPP index as small unextractable polymeric proteins (sUPP%). As expected and according to the data reported in Table 2, cookie sUPP% was significantly higher than that of Manitoba flour (24.62%), reflecting the differences emerged from the analysis of extractability. The highest sUPP% values were observed in CS and EMS40 cookies while the lowest values were calculated for RIS30 and DAD40 cookies. The effect of geographical origin on this index was not statistically significant. Instead, sUPP% decreased with the increase of the percentage of substitution. sUPP% values were in the following increasing order: RI, DA, EM cookies. According to these data, BSG composition and quantity can influence the organization of the protein network and, therefore, the aggregation events, probably as a result of their high fibre contents.

The results of Table 2 indicate that the control cookies had the lowest TPC and that, consistently with other researches (Heredia-Sandoval et al. 2020), BSG was responsible for the higher (from two to three times) phenolic contents of the enriched cookies, which were comprised between 4.36 ± 0.43 mg/g dw (RIS40) and 5.92 ± 0.03 mg/g dw (EMS30). These antioxidant concentrations are higher than those obtained in similar researches [around 50 mg/100 g fresh weight for Farcas et al. (2021)]. With the exception of the geographical origin, the single effects of the considered parameters were statistically significant. Concerning the single effect of BSG composition, the highest TPCs were detected in EM cookies. Regarding the single effect of the amount of BSG in the formulation, the TPC increased from 0 to 30% but not from 30 to 40%. Concerning antioxidant capacity, the control cookies showed the lowest Trolox concentrations (and the highest DPPH%) while the highest antioxidant capacity values (and the lowest DPPH%) were measured for EMD40 samples. The highest Trolox concentrations (and the lowest DPPH%) were detected in EM cookies. The antioxidant activity was positively affected by the percentage of BSG in the formulation due to the highest antioxidant activity of each type of BSG with respect to Manitoba flour.

Twelve phenolic compounds (5 phenolic acids, 4 flavanols, 2 flavonols, and a stilbene) were identified in BSG-enriched cookies (Table 3). Catechin, epicatechin, quercetin, and resveratrol were the main responsible for the biological activity of BSG-enriched cookies. In fact, such compounds are known as dietary supplements for their protective effects on DNA, proteins, and lipids from oxidative damage. Chlorogenic acid was detected only in the control cookies, which also showed the highest amounts of ferulic acid and kaempferol. Instead, control cookies did not contain compounds such as 4-hydroxybenzoic acid, catechin, epigallocatechin gallate, quercetin, and kaempferol, thus highlighting simpler phenolic profiles and lower antioxidant activity than the fortified samples (Table 3). Concerning the effects of the BSG composition, the addition of RI contributed higher contents of caffeic acid and epigallocatechingallate, while the enrichment with DA supplied higher amounts of *p*-coumaric acid and kaempferol. EM spent grain allowed to increase the concentrations of gallic acid, 4-hydroxybenzoic acid, ferulic acid, catechin, epicatechin, epigallocatechin, and quercetin of the corresponding cookies. Concerning the effects of the amount of BSG present in formulations, the highest concentrations of caffeic acid, catechin, epicatechin, quereting, and resveratrol were obtained at 30% substitution while the highest *p*-coumaric acid and epigallocatechingallate contents were detected at 40% replacement.

**Table 3:** Single and interactive effects of geographical origin, BSG composition, and amount of BSG in formulation on the phenolic profile (µg/g dw) of cookies

Cookies	Gallic acid	4-BA	Caffeic acid	Chlorogenic acid	Ferulic acid	*p*-Coumaric acid	Catechin	Epicatechin	EGC	EGCG	Quercetin	Resveratrol	Kaempferol
Interactive effects of geographical origin, BSG composition, BSG % in formulation													
CS	57.8 ± 0.4^i^	0.0 ± 0.0^a^	10.7 ± 0.0^a^	381.5 ± 19.9^b^	48.8 ± 0.0^g^	19.0 ± 0.1^ef^	0.0 ± 0.0^a^	165.3 ± 0.1^a^	38.6 ± 0.4^a^	0.0 ± 0.0a	0.0 ± 0.0a	0.0 ± 0.0^a^	477.9 ± 17.4^g^
RID30	31.2 ± 0.3^b^	16.4 ± 0.3^f^	117.9 ± 0.4^i^	0.0 ± 0.0^a^	47.5 ± 0.7^d^	18.2 ± 0.0^d^	3356. 9 ± 103.2^g^	2142.1 ± 12.5^bc^	37.1 ± 0.2^a^	2.9 ± 0.0^h^	30.5 ± 0.0^g^	32.7 ± 0.0^l^	125.4 ± 1.7^cd^
RID40	43.4 ± 0.9^g^	21.2 ± 2.2^g^	99.9 ± 1.4^d^	0.0 ± 0.0^a^	46.6 ± 0.0^c^	17.5 ± 0.0^c^	3518.0 ± 2.6^h^	2109.6 ± 14.3^b^	41.7 ± 0.4^a^	5.7 ± 0.1^k^	29.9 ± 0.0^cd^	32.0 ± 0.0^g^	128.2 ± 1.7^d^
RIS30	28.0 ± 0.3^a^	12.9 ± 0.9^e^	113.5 ± 1.8^h^	0.0 ± 0.0^a^	45.1 ± 0.1^a^	17.1 ± 0.0^a^	4250.7 ± 1.8^k^	2261.0 ± 19.2^d^	33.0 ± 0.3^a^	3.1 ± 0.3^h^	28.9 ± 0.0^b^	30.8 ± 0.0^b^	117.4 ± 0.7^bc^
RIS40	32.7 ± 0.2^c^	7.3 ± 0.0^c^	110.8 ± 3.1^g^	0.0 ± 0.0^a^	46.0 ± 0.1^b^	17.3 ± 0.0^ab^	2994.6 ± 18.0^f^	2153.7 ± 447.1^c^	37.3 ± 0.3^a^	1.6 ± 0.0^d^	29.1 ± 0.0^c^	31.1 ± 0.0^d^	115.3 ± 1.3^b^
DAD30	42.8 ± 0.6^g^	6.8 ± 0.1^c^	97.6 ± 0.0^cd^	0.0 ± 0.0^a^	46.0 ± 0.0^b^	18.2 ± 0.1^d^	2016.8 ± 5.0^c^	2113.6 ± 0.8^b^	37.9 ± 0.0^a^	2.4 ± 0.1^f^	29.9 ± 0.0^e^	30.8 ± 0.0^c^	133.5 ± 3.9^d^
DAD40	40.7 ± 0.3^f^	6.9 ± 0.2^c^	125.5 ± 0.9^k^	0.0 ± 0.0^a^	47.2 ± 0.0^d^	19.2 ± 0.1^ef^	1860.0 ± 12.0^b^	2777.9 ± 15.6^g^	40.1 ± 0.4^a^	2.7 ± 0.0^g^	31.2 ± 0.1^h^	32.2 ± 0.0^g^	149.4 ± 2.9^e^
DAS30	36.8 ± 0.3^d^	4.6 ± 0.2^b^	98.0 ± 2.0^cd^	0.0 ± 0.0^a^	48.4 ± 0.0^f^	19.4 ± 0.0^fg^	2353.7 ± 76.1^d^	2478.1 ± 11.6^f^	39.9 ± 0.1^a^	1.3 ± 0.1^c^	32.1 ± 0.1^l^	32.4 ± 0.0^i^	111.5 ± 1.7^b^
DAS40	43.3 ± 0.4^g^	9.7 ± 0.2^d^	103.7 ± 0.7^e^	0.0 ± 0.0^a^	48.1 ± 0.0^e^	19.0 ± 0.0^e^	2785.0 ± 50.0^e^	2498.8 ± 9.4^f^	66.0 ± 27.1^a^	4.4 ± 0.1^i^	31.6 ± 0.0^i^	32.3 ± 0.0^h^	174.2 ± 2.0^f^
EMD30	39.2 ± 0.5^e^	13.5 ± 0.7^e^	96.5 ± 0.1^cd^	0.0 ± 0.0^a^	49.3 ± 0.0^h^	18.2 ± 0.1^d^	3498.1 ± 164.4^h^	2724.2 ± 11.7^g^	16.2 ± 16.2^a^	2.5 ± 0.2^fg^	31.7 ± 0.1^k^	32.2 ± 0.0 g	97.5 ± 1.6^a^
EMD40	71.4 ± 0.4^k^	25.3 ± 1.1^h^	94.2 ± 0.1^b^	0.0 ± 0.0^a^	48.5 ± 0.0^f^	17.5 ± 0.4^bc^	3900.8 ± 188.9^i^	2274.5 ± 44.3^d^	156.0 ± 156.0^b^	1.4 ± 0.0^c^	32.5 ± 0.0^c^	31.9 ± 0.0^e^	128.3 ± 0.9^d^
EMS30	39.0 ± 0.2^e^	20.7 ± 0.5^g^	106.8 ± 2.1^f^	0.0 ± 0.0^a^	49.3 ± 0.0^h^	19.0 ± 0.1^ef^	4444.2 ± 57.0^l^	2732.5 ± 21.5^g^	38.8 ± 0.2^a^	1.0 ± 0.1^b^	31.9 ± 0.0^b^	32.6 ± 0.0^l^	99.7 ± 0.2^a^
EMS40	56.9 ± 0.3^h^	31.0 ± 1.0^k^	92.8 ± 0.8^b^	0.0 ± 0.0^a^	46.2 ± 0.1^b^	18.1 ± 0.1^d^	4631.0 ± 48.5^m^	2328.6 ± 12.3^e^	36.9 ± 0.6^a^	1.9 ± 0.2^e^	30.1 ± 0.0^e^	31.1 ± 0.0^d^	118.3 ± 3.4^bd^
*Significance*	*	*	*	*	*	*	*	*	*	*	*	*	*
Single effect of geographical origin													
D(aunia)	44.8^b^	15.0^b^	105.3^b^	0.0^a^	47.5^b^	18.1^a^	3025.1^a^	2356.9^a^	54.8^a^	2.9^b^	30.9^b^	32.0^b^	127.0^b^
S(alento)	39.4^a^	14.3a	104.2^a^	0.0^a^	47.2^a^	18.3^b^	3576.5^b^	2408.8^b^	41.9^a^	2.2^a^	30.6^a^	31.7^a^	122.7^a^
*Significance*	*	*	*	ns	*	*	*	*	ns	*	*	*	*
Single effect of BSG composition													
RI	37.8^a^	14.4^b^	110.5^c^	0.0^a^	46.3^a^	17.5^a^	3530.0^b^	2166.6^a^	37.2^a^	3.3^c^	29.6^a^	31.7^a^	121.6^b^
DA	40.9^b^	7.0^a^	106.2^b^	0.0^a^	47.4^b^	18.9^c^	2253.8^a^	2467.1^b^	46.0^a^	2.7^b^	31.2^b^	31.9^a^	142.1^c^
EM	51.7^c^	22.6^c^	97.6^a^	0.0^a^	48.3^c^	18.2^b^	4118.5^c^	2514.9^c^	61.9^a^	1.7^a^	31.6^c^	31.9^a^	111.0^a^
*Significance*	*	*	*	ns	*	*	*	*	ns	*	*	ns	*
Single effect of BSG % in formulation													
0	57.8^c^	0.0^a^	106.6^a^	^381.4b^	48.8^c^	18.1^a^	0^a^	1653.0^a^	38.6^a^	0.0^a^	0.0^a^	0.0^a^	477.9^c^
30	36.2^a^	12.5^b^	104.4^b^	0.0^a^	47.6^b^	18.4^b^	3320.1^b^	2408.6^c^	33.8^a^	2.2^a^	30.8^c^	31.9^b^	114.2^a^
40	48.1^b^	16.9^c^	105.0^b^	0.0^a^	47.1^a^	19.0^c^	3281.5^b^	2357.1^b^	63.0^a^	2.9^b^	30.7^b^	31.8^b^	135.6^b^
*Significance*	*	*	*	*	*	*	*	*	ns	*	*	*	*

In column, different letters indicate significant differences at *p* < 0.05 by LSD multiple range test

nd: not detected

the asterisks indicate significant differences at *p* < 0.05 by LSD multiple range test

ns: not significant

4-HBA: 4-hydroxybenzoic acid; EGC: Epigallocatechin; EGCG: Epigallocatechingallate

### Sensory characteristics of the BSG-enriched cookies

Biscuits/cookies are not staple foods, which is why the willingness to consume their functional version made with agri-food by-products is more linked to the sensory characteristics of the products rather than to their nutritional contribution or the potential environmental benefits (Crofton & Scannell 2020). Data concerning the effects of geographical origin, formulation, and amount of BSG on the sensory profile of cookies are listed in Table 4.

**Table 4:** Single and interactive effects of geographical origin, BSG composition, and amount of BSG in formulation on the sensory profile of cookies

Cookies	Colour intensity	Flavours						Tastes		Tactile characteristics and Texture					Overall Quality
		Overall Intensity	Freshly baked	Wheat	Malty	Toasty	Hazelnut	Sweetness	Bitterness	Astringency	Hardness	Crunchiness	Fibrousness/Graininess	Friability	
Interactive effects of geographical origin, BSG composition, BSG % in formulation															
CS	4.0 ± 0.0^a^	4.7 ± 0.6^a^	2.7 ± 1.5^bc^	3.0 ± 0.0^e^	2.3 ± 1.2^ab^	0.3 ± 0.3^a^	1.0 ± 1.0^a^	5.0 ± 0.0^a^	1.3 ± 1.2^b^	0.7 ± 0.6^b^	4.0 ± 1.0^a^	6.3 ± 1.5^bc^	2.7 ± 2.3^b^	4.7 ± 1.2^a^	6.0 ± 1.0^b^
RID30	6.3 ± 0.6^b^	6.0 ± 0.0^b^	7.0 ± 0.0^g^	3.0 ± 0.0^e^	5.0 ± 0.0^e^	3.0 ± 0.0^d^	3.7 ± 0.6^d^	5.7 ± 0.6^b^	1.0 ± 0.0^b^	0.0 ± 0.0^a^	6.7 ± 0.6^d^	7.7 ± 0.6^de^	5.0 ± 0.0^c^	8.0 ± 0.0^de^	7.7 ± 0.6^c^
RID40	7.7 ± 0.6^c^	6.0 ± 0.0^b^	3.3 ± 0.6^ce^	2.3 ± 0.6^cd^	4.0 ± 0.0^d^	2.0 ± 0.0^c^	1.0 ± 0.0^a^	7.0 ± 0.0^e^	1.0 ± 0.0^b^	0.0 ± 0.0^a^	6.0 ± 0.0^c^	8.0 ± 0.0^e^	2.0 ± 0.0^ab^	7.7 ± 0.6^d^	8.7 ± 0.6^d^
RIS30	7.7 ± 0.6^c^	7.0 ± 0.0^cd^	4.0 ± 0.0^e^	2.0 ± 0.0^c^	4.0 ± 0.0^d^	3.0 ± 0.0^d^	2.0 ± 0.0^b^	6.0 ± 0.0^bc^	0.0 ± 0.0^a^	0.0 ± 0.0^a^	6.0 ± 0.0^c^	9.0 ± 0.0^f^	2.0 ± 0.0 ^ab^	8.7 ± 0.6^e^	8.7 ± 0.6^d^
RIS40	8.0 ± 0.0^cd^	7.3 ± 0.6^de^	3.0 ± 0.0^cd^	3.0 ± 0.0^e^	3.0 ± 0.0^bc^	5.0 ± 0.0^f^	2.0 ± 0.0^b^	6.7 ± 0.6^de^	3.0 ± 0.0^d^	0.0 ± 0.0^a^	5.0 ± 0.0^b^	6.0 ± 0.0^b^	2.0 ± 0.0 ^ab^	5.0 ± 0.0^a^	6.0 ± 0.0^b^
DAD30	8.0 ± 0.0^cd^	7.7 ± 0.6^e^	8.0 ± 0.0^h^	2.7 ± 0.6^de^	2.7 ± 0.6^ab^	3.3 ± 0.6^c^	3.7 ± 0.6^d^	6.3 ± 0.6^cd^	0.0 ± 0.0^a^	0.0 ± 0.0^a^	7.0 ± 0.0^d^	8.0 ± 0.0^e^	1.0 ± 0.0^a^	8.0 ± 0.0^de^	8.3 ± 0.6^cd^
DAD40	8.7 ± 0.6^d^	6.7 ± 0.6^c^	4.0 ± 0.0^e^	2.0 ± 0.0^c^	2.0 ± 0.0^a^	2.0 ± 0.0^c^	1.0 ± 0.0^a^	7.0 ± 0.0^e^	0.0 ± 0.0^a^	0.0 ± 0.0^a^	6.0 ± 0.0^c^	6.0 ± 0.0^b^	1.0 ± 0.0 ^a^	8.7 ± 0.6^e^	8.0 ± 0.0^cd^
DAS30	8.7 ± 0.6^d^	6.0 ± 0.0^b^	3.7 ± 0.6^de^	3.0 ± 0.0^e^	3.0 ± 0.0^bc^	2.0 ± 0.0^c^	3.0 ± 0.0^c^	7.7 ± 0.6^f^	0.0 ± 0.0^a^	0.0 ± 0.0^a^	5.0 ± 0.0^b^	5.0 ± 0.0^a^	1.0 ± 0.0 ^a^	5.3 ± 0.6^ab^	8.0 ± 0.0^cd^
DAS40	8.7 ± 0.6^d^	7.3 ± 0.6^de^	3.7 ± 0.6^de^	2.0 ± 0.0^c^	2.0 ± 0.0^a^	4.0 ± 0.0^e^	3.0 ± 0.0^c^	7.7 ± 0.6^f^	0.0 ± 0.0^a^	0.0 ± 0.0^a^	6.0 ± 0.0^c^	8.0 ± 0.0^e^	1.0 ± 0.0 ^a^	6.3 ± 0.6^c^	8.3 ± 0.6^cd^
EMD30	8.0 ± 0.0^cd^	7.0 ± 0.0^cd^	5.3 ± 0.6^f^	0.0 ± 0.0^s^	3.7 ± 0.6^cd^	1.0 ± 0.0^b^	1.0 ± 0.0^a^	5.7 ± 0.6^b^	1.0 ± 0.0^b^	0.0 ± 0.0^a^	7.0 ± 0.0^d^	7.0 ± 0.0^cd^	5.0 ± 0.0^c^	7.3 ± 0.6^d^	6.0 ± 0.0^b^
EMD40	8.3 ± 0.6^cd^	7.3 ± 0.6^de^	1.0 ± 0.0^a^	1.0 ± 0.0^b^	3.7 ± 0.6^cd^	3.0 ± 0.0^d^	3.0 ± 0.0^c^	6.0 ± 0.0^bc^	2.0 ± 0.0^c^	0.0 ± 0.0^a^	8.0 ± 0.0^e^	8.0 ± 0.0^e^	5.0 ± 0.0^c^	7.3 ± 0.6^d^	6.0 ± 0.0^b^
EMS30	8.0 ± 0.0^cd^	7.0 ± 0.0^cd^	2.0 ± 0.0^b^	0.0 ± 0.0^a^	2.3 ± 0.6^ab^	3.0 ± 0.0^d^	3.0 ± 0.0^c^	6.0 ± 0.0^bc^	1.0 ± 0.0^b^	0.0 ± 0.0^a^	8.0 ± 0.0^e^	8.0 ± 0.0^e^	5.0 ± 0.0^c^	7.3 ± 0.6^d^	6.0 ± 0.0^b^
EMS40	8.7 ± 0.6^d^	7.0 ± 0.0^cd^	4.0 ± 0.0^e^	0.0 ± 0.0^a^	3.7 ± 0.6^cd^	2.0 ± 0.0^c^	3.0 ± 0.0^c^	8.0 ± 0.0^f^	2.0 ± 0.0^c^	0.0 ± 0.0^a^	7.0 ± 0.0^d^	6.0 ± 0.0^b^	3.0 ± 0.0^b^	6.0 ± 0.0^bc^	5.0 ± 0.0^a^
*Significance*	*	*	*	*	*	*	*	*	*	*	*	*	*	*	*
Single effect of geographical origin															
D(aunia)	7.8^a^	6.8^a^	4.8^b^	1.8^b^	3.5^b^	2.4^a^	2.2^a^	6.3^a^	0.8^a^	0.0^a^	6.8^b^	7.4^b^	3.2^b^	7.8^b^	7.0^a^
S(alento)	8.3^a^	6.9^a^	3.4^a^	1.7^a^	3.0^a^	3.2^b^	2.7^b^	7.0^b^	1.0^b^	0.0^a^	6.2^a^	7.0^a^	2.3^a^	6.4^a^	7.4^b^
*Significance*	ns	ns	*	*	*	*	*	*	*	ns	*	*	*	*	*
Single effect of BSG composition															
RI	7.4^a^	6.6^a^	4.3^b^	2.6^b^	4.0^c^	3.2^c^	2.2^a^	6.3^a^	1.2^b^	0.0^a^	5.9^a^	6.7^a^	2.7^b^	7.3^a^	7.7^b^
DA	8.5^b^	6.9^b^	4.8^c^	2.4^b^	2.4^a^	2.8^b^	2.7^b^	7.2^b^	0.0^a^	0.0^a^	6.0^a^	7.6^c^	1.0^a^	7.1^a^	8.2^c^
EM	8.2^b^	7.1^b^	3.1^a^	0.2^a^	3.3^b^	2.2^a^	2.5^b^	6.4^a^	1.5^b^	0.0^a^	7.5^b^	7.2^b^	4.5^c^	7.0^a^	5.7^a^
*Significance*	*	*	*	*	*	*	*	*	*	ns	*	*	*	ns	*
Single effect of BSG % in formulation															
0	4.0^a^	4.7a	2.7^a^	3.0^b^	2.3^a^	0.3^a^	1.0^a^	5.0^a^	1.3^b^	0.7^b^	4.0^a^	6.3^a^	2.7^ab^	4.7^a^	6.0^a^
30	7.8^b^	6.8^b^	5.0^b^	1.8^a^	3.4^c^	2.6^b^	2.7^c^	5.2^b^	0.5^a^	0.0^a^	6.6^b^	7.4^c^	3.2^b^	7.4^c^	7.4^b^
40	8.3^c^	6.9^b^	3.2^a^	1.7^a^	3.1^b^	3.0^c^	2.2^b^	7.1^c^	1.3^b^	0.0^a^	6.3^b^	7.0^b^	2.3^a^	6.8^b^	7.0^b^
*Significance*	*	*	*	*	*	*	*	*	*	*	*	*	*	*	*

In column, different letters indicate significant differences at *p* < 0.05 by LSD multiple range test

the asterisks indicate significant differences at *p* < 0.05 by LSD multiple range test

ns: not significant

Consistently with the colorimetric data, the addition of BSG significantly increased the cookie colour intensity from 4 (control) to values comprised between 6.3 (RID30) and 8.7 (DAD40, DAS30, DAS40, EMS40), causing a sensory detectable browning that significantly rose with the substitution level.

Control cookies achieved the lowest scores for the overall flavour intensity and toasty and hazelnut smells. The overall flavour intensity was affected by BSG composition because it was the highest in DA. It was also positively affected by the addition of BSG in formulation from 0 to 30% replacement while the BSG increase to 40% did not determine a corresponding increase of that variable. The toasty smell was the highest in RI-enriched cookies and increased with the amount of BSG in cookies formulation. The highest intensity of freshly baked, malty, and hazelnut smells was perceived at a 30% substitution. The freshly baked smell was higher in DA spent grain probably as a result of the presence of 3-methyl-1 butanol (De Flaviis et al. 2021). The wheat smell was greatly perceived in control cookies and in cookies containing RI and DA spent grains while it was not perceived in EM-products.

Sweetness intensity ranged from 5.0 ± 1.0 (CS) to 8.0 ± 0.0 (EMS40). It was intensified by the presence of DA spent grains and was also positively affected by the percentage of substitution. Bitterness was not perceived in DA cookies, probably because covered by their sweetness, and was minimized at 30% substitution while astringency was perceived only in the control cookies, perhaps as a consequence of their lower sweeteness.

The addition of BSG caused significant increase of hardness, which varied from 4.0±1.0 of control cookies up to values comprised between 5.0±0.0 (RIS40, DAS30) and 8.0±0.0 (EMD40, EMS30) of the enriched samples. This behaiour was explained taking into account the absorption of water by BSG proteins and fibres (Petrovic et al. 2017). Hardness was affected by composition of BSG since it was increased by the presence of EM spent grains (having the highest fibre content). Ita also increased with the increasing substitution level.

A mean crunchiness score of 6.3 was assigned to the control cookies while enriched cookies ranged between 5.0 of DAS30 to 9.0 of RIS30 samples. Contrarily to the findings of Laguna et al. (2014), crunchiness was not depressed by the addition of BSG, probably as a result of the low water-binding capacity of the spent grain fibrils (Piteira et al. 2006), which were not able to create a compact structure.

BSG composition and amount significantly affected the cooking fibrousness, which ranged from 1.0±0.0 of DA-cookies to 5.0±0.0 of RID30, EMD30, EMD40, and EMS30 samples. More in depth, EM spent grain contributed the highest fibrousness, followed by RI. Surprisingly, a greater fibrousness was highlighted at 30% substitution instead of 40%. These results can be only partially attributed to the fibre content of the BSG. Probably they were influenced by an uneven distribution of fibre clusters (Piteira et al. 2006).

Friability ranged from 4.7 ± 1.2 (control) to 8.7 ± 0.6 of RIS30 and DAD40 samples. The enriched cookies were more brittle than the control due to the effects of the high quantity of insoluble fibres supplied by spent grains. Having a low water-binding capability and being relatively long, these insoluble fibres formed weaker clusters and determine a certain reduction of the wheat flour extensibility (Piteira et al. 2006).

In our study, the overall quality ranged from 5.0 ± 0.0 (EMS0) to 8.7 ± 0.6 (RID40 and RIS30). The overall rating was affected by BSG formulation, with the best judgment attributed to DA and RI cookies. The trained panellists also accorded their preferences to cookies with 30% BSG in their formulation. Excepted for EMS40 samples, all the enriched cookies obtained equal or higher overall quality scores than control. This represented a good result since Kim et al. (2001) reported a reduction of the overall sensory score in products containing more than 15% BSG.

The effects of geographical origin on the sensory characteristics of cookies deserve a separate discussion. They are certainly to be attributed to the substantial differences in soil and climate conditions between the two location and therefore it is difficult to interpret (Cotecchia et al. 2014; L’Abate et al. 2015). Panellists attributed higher scores to toasty and hazelnut flavour and to sweetness and bitterness of cookies produced with BSG from S(alento). Instead, cookies produced with BSG from D(aunia) received the highest scores for intensity of freshly baked, wheat, and malty flavours, and for all tactile characteristics (except astringency). Furthermore, cookies produced with BSG from S(alento) obtained the highest overall sensory scores. Based on the panellist responses, the various visual, olfactory and gustatory characteristics had different weights on the overall sensory quality.

### Relationship among physical, chemical, and sensory characteristics of cookies

**Fig. 2:**
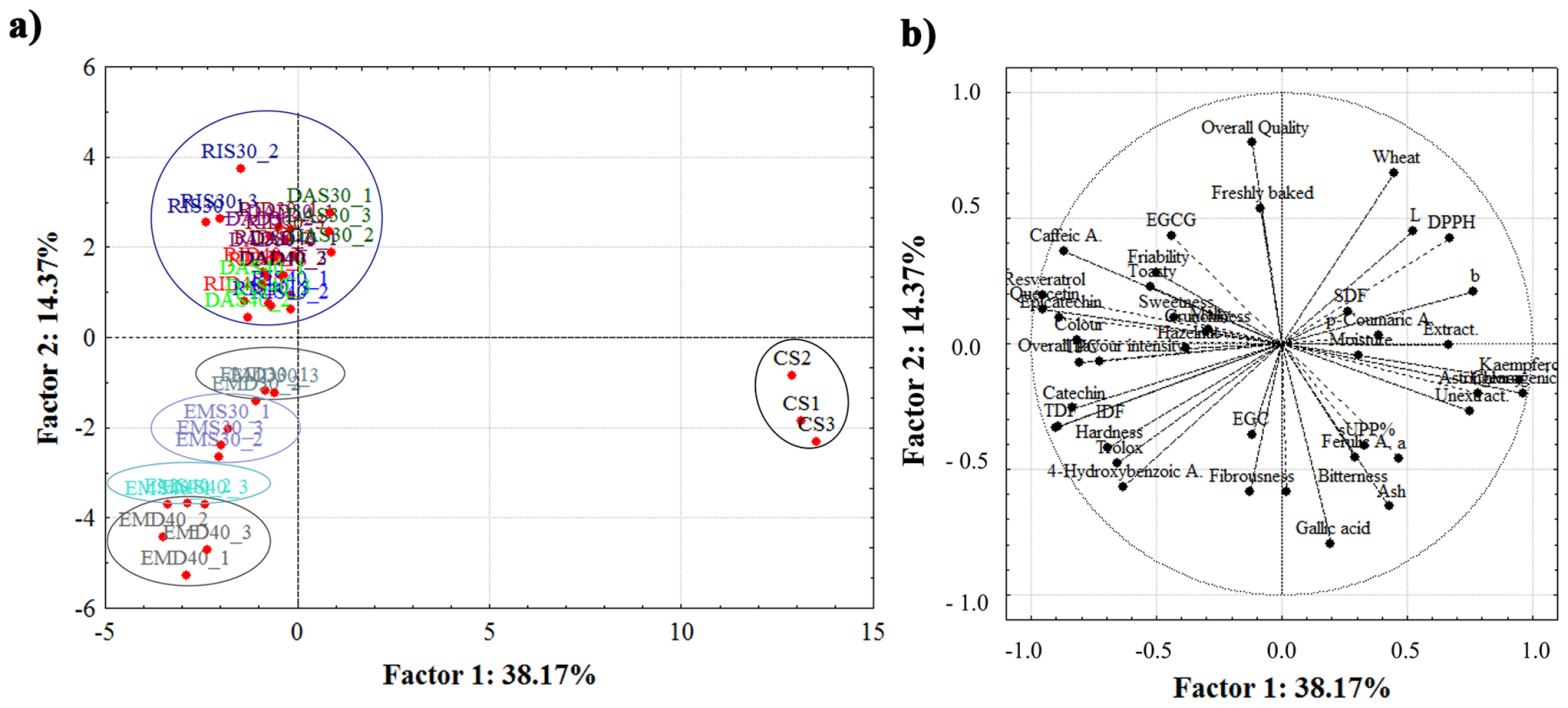
Principal Component Analysis of control cookies and cookies enriched with brewers’ spent grain: projections of (**a**) the samples and (**b**) their physical, chemical, and sensory profiles on the factorial plane

Figure 2 shows the PCA bidimensional map of control and enriched cookies (a) and the corresponding analytical profile (b). The first two principal components explain 52.5% of the total variance. Figure 2a shows a clear separation between control and all enriched cookies, characterized by positive and negative values of the factor 1, respectively. Control cookies were associated to high percentages of SDS-extractable and unextractable proteins, high contents of chlorogenic acid and kaempferol, and high astringency (Fig. 2a and b). The factor 2 allowed to homogeneously group the four types of EM cookies based on the different contents of 4-hydroxybenzoic acid, catechin, and epigallocatechin (Fig. 2a and b). Cookies enriched with RI and DA spent grains constituted a single large indistinguishable grouping, placed in the quadrant characterized by the highest overall quality scores (also higher than that of control cookies). These high overall quality scores were probable related to the high intensity of fresh baked flavour and to the low hardness and fibrousness of RI- and DA-cookies.

The bivariate Pearson correlation analysis (Table S1) revealed that:

the colorimetric index b* was significantly and positively correlated with L* (*R* = 0.82), chlorogenic acid and kaempferol contents (0.72 and 0.74), and negatively correlated with the insoluble dietary fibre (-0.72) and quercetin content (-0.70);

the total dietary fibre was positively correlated with IDF (1.00), total phenolic content (0.78), Trolox concentrations (0.71), concentrations of 4-hydroxybenzoic acid, caffeic acid, catechin, epicatechin, quercetin, and resveratrol (0.71–0.85), and inversely correlated with the chlorogenic acid content (-0.81);

the SDS-unextractable protein fraction was positively correlated with the chlorogenic acid content (0.75) and inversely correlated with the concentrations of caffeic acid, quercetin, and resveratrol (-0.71; -0.73).

The identification of significant correlations between colorimetric indices, dietary fibre, protein fractions, individual phenolic content, total phenolic content, and antioxidant activity allowed an indirect estimation of the compounds that can contribute to the food functional characteristics. According to these relationships, phenolic fraction, products of the Maillard reactions, and dietary fibres contributed to the cookies antioxidant activity. The contribution of the dietary fbres was probably due to the non-extractable polyphenols i.e. polyphenols bound to dietary fibre (Wang et al. 2024). The negative relationships among unextractable proteins and some phenolic compounds was probably a consequence of phenolic-protein interactions (Welc et al. 2022).

## Conclusion

Considering the importance of antioxidants and dietary fibres to human health, the consumption of BSG-enriched cookies can have the potential to exert remarkable benefits.

Cookies fortified with BSG showed higher contents of soluble and insoluble fibres, phenolic compounds, antioxidant activity and better sensory characteristics than the control ones. Cookies fortified with 30% BSG flour showed good ash, dietary fibre, and total phenolic contents and received higher overall quality scores if produced with DA or RI spent grain in their formulation. Even in absence of significant correlations, those cookies were characterized by intense brown colour, low bitterness, intermediate hardness, and high intensity of freshly baked smell, sweetness, crunchiness, and friability. Regarding the effects of geographical origin, this factor significantly influenced some physical, chemical, and sensory parameters. More in depth, cookies from S(alento) had lower ash content, and higher moisture, insoluble dietary fibre contents, and overall sensory quality than the cookies obtained with the cereals cultivated in Daunia area.

## Electronic supplementary material

Below is the link to the electronic supplementary material.

Supplementary Material 1

Supplementary Material 2

## Data Availability

Raw data on which the manuscript is based are available.

## Abbreviations

BSG: Dried Brewers’ Spent Grain Flours
C: Control Cookies
RI: BSG deriving from mashing of a mixture of 65% barley malt and 35% unmalted soft wheat cv. Risciola during brewing of craft Belgian style white beers
DA: BSG deriving from mashing of a mixture of 65% barley malt and 35% unmalted durum wheat cv. Dauno III brewing of craft Belgian style white beers
EM: BSG deriving from mashing of a mixture of 65% barley malt and 35% unmalted emmer
30 and 40: Percentages of substitution of the Manitoba flour with one of the BSG flours in cookie production
EVOO: Extra-Virgin Olive Oil
TPC: Total Phenolic Content
sUPP%: Percentage of small unextractable polymeric proteins
TDF: Total Dietary Fibres
SDF: Soluble Dietary Fibres
IDF: Insoluble Dietary Fibres
