# Supplementary figures and images for: Application of a multivariate approach to the study of chemometric and sensory profiles of cookies fortified with brewers’ spent grain
Source: J Food Sci Technol. 2024 Aug 23;62(4):738–50. doi: 10.1007/s13197-024-06064-3 (PMC11914526; doi:10.1007/s13197-024-06064-3)

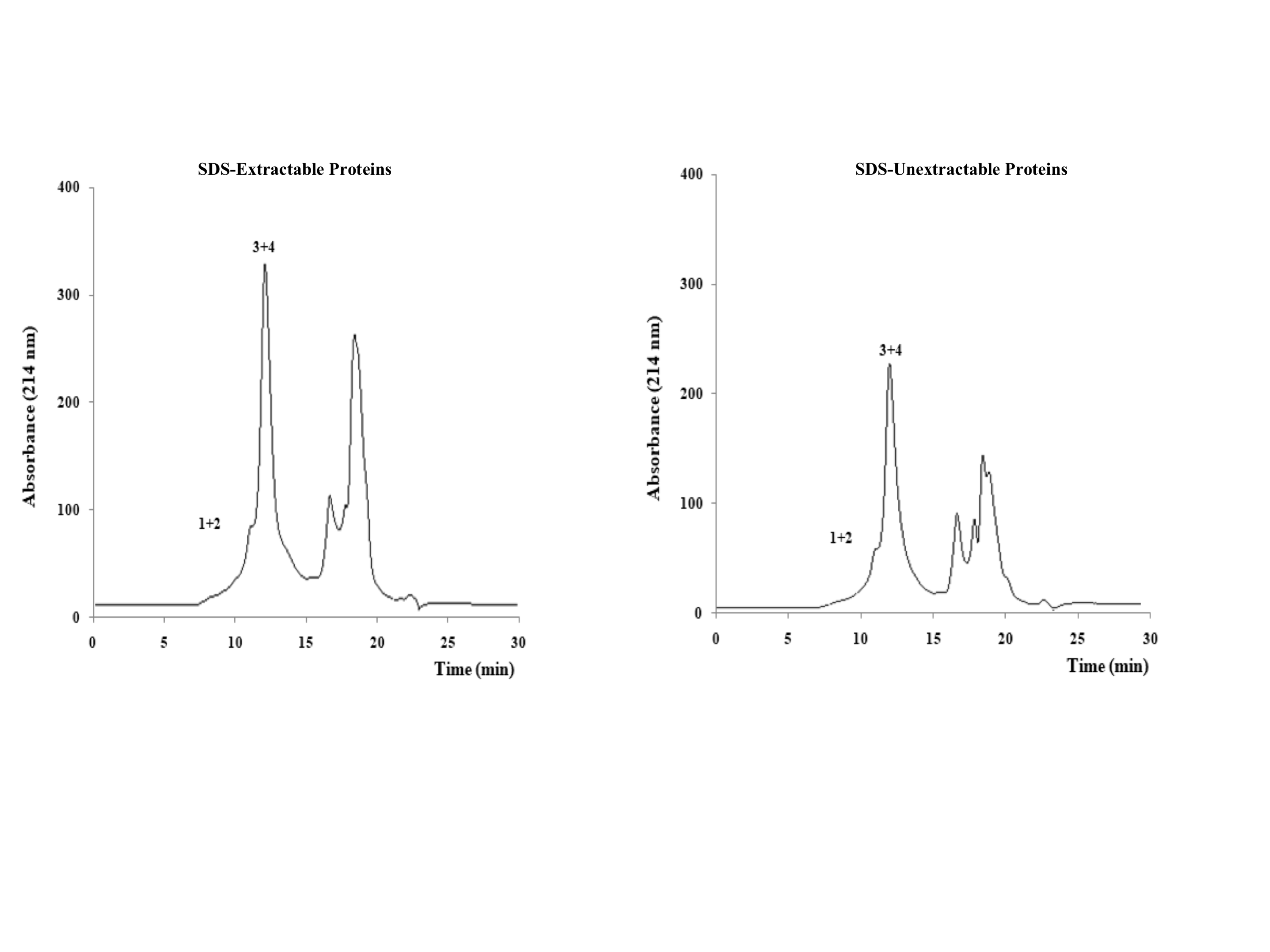

Supplement: Supplementary file 1 — Supplementary Material 1 [file 13197_2024_6064_MOESM1_ESM.tiff]
